# Isolation and characterization of vaginal *Lactobacillus* spp. in dromedary camels (*Camelus dromedarius*): in vitro evaluation of probiotic potential of selected isolates

**DOI:** 10.7717/peerj.8500

**Published:** 2020-02-05

**Authors:** Wael M. El-Deeb, Mahmoud Fayez, Ibrahim Elsohaby, Ibrahim Ghoneim, Theeb Al-Marri, Mahmoud Kandeel, Magdy ElGioushy

**Affiliations:** 1 Department of Clinical Sciences, College of Veterinary Medicine, King Faisal University, Al-Ahsa, Saudi Arabia; 2 Department of Veterinary Medicine, Infectious Diseases and Fish Diseases, Faculty of Veterinary Medicine, Mansoura University, Mansoura, Egypt; 3 Ministry of Agriculture, Al-Ahsa Veterinary Diagnostic Laboratory, Al-Ahsa, Saudi Arabia; 4 Veterinary Serum and Vaccine Research institute, Ministry of Agriculture, Cairo, Egypt; 5 Department of Animal Medicine, Faculty of Veterinary Medicine, Zagazig University, Zagazig, Sharkia Governorate, Egypt; 6 Department of Health Management, Atlantic Veterinary College, University of Prince Edward Island, Charlottetown, Canada; 7 Department of Theriogenology, Faculty of Veterinary Medicine, Cairo University, Giza, Egypt; 8 Department of Biomedical Sciences, College of Veterinary Medicine, King Faisal University, Al-Ahsa, Saudi Arabia; 9 Department of Pharmacology, Faculty of Veterinary Medicine, Kafrelsheikh University, Kafr Elsheikh, Egypt; 10 Department of Animal Medicine, Faculty of Veterinary Medicine, Aswan University, Aswan, Egypt

**Keywords:** *Lactobacillus plantarum*, *Lactobacillus fermentum*, Autoaggregation, Coaggregation, Antimicrobial, Uterus, Infection, 16S rRNA, Endometritis, *Lactobacillus rhamnosus*

## Abstract

*Lactobacillus* spp. is one of the beneficial lactic acid producing microbiota in the vagina, which is important for a healthy vaginal environment. However, little is known about vaginal *Lactobacillus* in dromedary camels (*Camelus dromedarius*). Therefore, this study aimed to isolate vaginal lactic acid bacteria (LAB) in dromedary camels and to study the probiotic potential of selected isolates. A total of 75 vaginal swabs were collected from pluriparous, non-pregnant, non-lactating dromedary camels. The LAB were isolated using deMan, Rogosa and Sharpe broth and agar media. Suspected LAB isolates were subjected to catalase testing and Gram staining and examined for indole production, nitrate reduction, hemolytic activity, cell surface hydrophobicity, auto- and coaggregation, antibacterial activity and characterized by 16S rRNA amplification and sequencing. Eighteen LABs were isolated from the 75 vaginal swabs. Among the 18 LAB isolates, six were *Lactobacillus plantarum*, eight were *Lactobacillus fermentum*, and four were *Lactobacillus rhamnosus*. None of the LAB isolates was hemolytic and only four LAB were H_2_O_2_ producing. The percentage of hydrophobicity ranged from 0% to 49.6%, 0% to 44.3% and 0% to 41.6% for hexadecane, xylene and toluene, respectively. All isolates showed higher (*P* < 0.05) autoaggregation after 24 h of incubation compared to 4 h. Furthermore, all LAB showed higher coaggregation (*P* < 0.05) and antimicrobial activity toward *Staphylococcus aureus* than to *Escherichia coli*. All LAB isolates were vancomycin resistant and sensitive to streptomycin, erythromycin, kanamycin and chloramphenicol. Only, three LAB isolates were resistant to tetracycline. The dromedary camel vaginal LAB isolates exhibited varying degrees of in vitro probiotic properties tested in this study and showed promising activity against the most common bacterial causes of endometritis in dromedary camels. Further investigation of the in vivo effect of these isolates is warranted.

## Introduction

Bacteria colonizing the reproductive tract of the she-camel (*Camelus dromedarius*) have been reported to be the main causes of reproductive problems (Wernery & Wernery, 1992; Wernery & Kumar, 1994; Tibary et al., 2006; Ali et al., 2010). Camel endometritis is a major cause of infertility of camels in Saudi Arabia. About 57.1% of cases with reproductive disorders were due to metritis and endometritis (Ali et al., 2010). The most common uterine bacterial isolates in dromedaries are *Campylobacter* spp., *Brucella* spp., *Coxiella burnetii*, *Salmonella* spp., *Chlamydia* spp., *Escherichia coli*, *Pseudomonas* and *Staphylococcus aureus* (Al-Afaleq et al., 2012; Mshelia1 et al., 2014; Khalafalla et al., 2017; El-Deeb et al., 2019). Owing to the diversity of these pathogens, a single antibiotic program might not be sufficient due to the bacterial resistance and the broad range of bacterial species. Therefore, probiotics have been introduced as a novel strategy for the treatment program and preventive measures of reproductive tract diseases (Reid & Burton, 2002). Different definitions have been reported for probiotics based on the mechanisms and site of action, delivery format and method, and host. However, the internationally recognized meaning of probiotics is “live microorganisms that, when administered in adequate amounts, confer a health benefit on the host” (FAO/WHO, 2001; Sanders, 2008).

Vaginal microbiota protects the host against pathogen colonization. Lactic acid bacteria (LAB) are the most abundant vaginal microbiota (Mårdh, 1991; Anukam et al., 2006; Pires et al., 2016) and comprise an order of diverse groups of Gram-positive bacteria that have a high tolerance for low pH levels (Van Geel-Schutten et al., 1998; De Vuyst & Leroy, 2007). Lactobacilli impair pathogen colonization by occupying the adhesion site of the vaginal epithelium in addition to producing antimicrobial compounds including hydrogen peroxide, lactic acid and bacteriocin-like compounds (Aroutcheva et al., 2001; Mokoena, 2017).

Together with bifidobacteria, LAB have been the most frequently investigated probiotics over the last 10 years. These probiotic microorganisms need to be assessed for the existence of probiotic properties, including antimicrobial activity against particular pathogens and the production of antimicrobial compounds (Tachedjian et al., 2017). The LAB isolated from various environments and fermented foods have a long history of safe use as probiotics (Naidu, Bidlack & Clemens, 1999; Saarela et al., 2000). Furthermore, vaginal LAB have been isolated in women (Fraga et al., 2008) and a broad range of domesticated animals including cattle (Otero et al., 2000), horses (Newcombe, 1978; Hinrichs et al., 1988) and pigs (Bara et al., 1993), and are highly valued for their probiotic properties. Consequently, the she-camel vagina may spontaneously constitute a reservoir for new LAB strains with possible probiotic properties. A previous study investigated the probiotic features of LAB from camel milk (Abushelaibi et al., 2017). However, to the best of the authors’ knowledge, no study has explored the probiotic potential of vaginal LAB isolates from camels. As uterine infections are the most recognized causative factors for camelids infertility (Johnson, 1989; Wernery & Kumar, 1994; Tibary et al., 2006; Khalafalla et al., 2017), lactobacillus-based probiotics may provide a viable approach to alleviate this fertility problem.

Lactobacillus-based probiotics are contrarily impacted by inflammatory condition in the uterus caused by challenging bacteria resulting in better fertility rates (Peter et al., 2018). Furthermore, LAB isolates can be optimized to get a clinically important product in replacement of antibiotics programs. Since raising concerns of antibiotics use due to their residues in animal products and their health hazards (Beyene, 2016; Ghoneim et al., 2017), probiotics are the best replacement with an anticipated high antimicrobial efficacy. Thus, the present study aimed to isolate vaginal LAB from dromedary camels (*C. dromedarius*) and to investigate their probiotic potential.

## Materials and Methods

### Animals and sampling

Dromedary she-camels (*n* = 75) aged 6–15 years, from the farm of the Camel Research Center (25° 23′N 49° 36′E), King Faisal University were sampled during the breeding season between November 2017 and April 2018. The camels were pluriparous, non-pregnant and non-lactating. All camels were clinically healthy and had a history of good fertility. The she-camels were kept under standard feeding and management practices. After proper cleaning and disinfecting of the vulvar area, vaginal swabs (*n* = 75) were collected from the lateral vaginal walls using a sterile, long-handled cotton swab (EQUIVET uterine culture swab, Kruuse, Denmark). Each swab was kept in two mL of *Lactobacillus* deMan, Rogosa and Sharpe (MRS) Broth (BD-Difco) (Rogosa, Mitchell & Wiseman, 1951) and transported in a cooler box to the laboratory. Deanship of Scientific Research provided full approval for this research (No. 7/B/9512).

### Lactobacillus isolation and characterization

The swab samples were streaked on MRS agar (BD-Difco) (Rogosa, Mitchell & Wiseman, 1951) and incubated anaerobically at 37 °C for 48 h. Suspected colonies were cultivated twice on MRS agar for purification. Isolates were presumptively identified as LAB based on the phenotypic characteristics (Kandler & Weiss, 1986) and were stored in milk yeast extract (13% fat-free milk, 1% yeast extract) containing 20% glycerol (vol./vol.) at −80 °C for later biochemical characterizations.

Suspected isolates were subjected to Gram staining and catalase testing and examined for nitrate reduction and indole production. For evaluation of the ability of LAB to grow at different pH, cultures were grown in MRS broth at 37 °C overnight, and sub-cultured in 10 mL of fresh MRS broth adjusted to different pH values (3.0, 3.5, 4.0, 4.5 and 7.0) with hydrochloric acid (3.0 M) according the methods described previously (Hydrominus et al., 2000). Hemolytic activity was also evaluated by streaking the LAB isolates on 5% sheep blood agar plates (Oxoid, Basingstoke, UK), followed by incubation at 37 °C for 48 h (Maragkoudakis et al., 2009). Sugar fermentation was identified on API 50 CH strips (BioMérieux Vitec, Inc., Lyon, France).

The ability of LAB to produce hydrogen peroxide (H_2_O_2_) was qualitatively assessed by streaking the isolates on MRS agar containing tetramethyl–benzidine (TMB) and horseradish peroxidase (Sigma–Aldrich, Seelze, Germany). The plates were incubated anaerobically at 37 °C for 48 h. Colonies that had produced H_2_O_2_ appeared dark blue (Rodriguez et al., 2011). All bacteriological culture media were subjected to quality control before use (Weenk et al., 1992) and uncultured media were included with each test as a negative control to ensure the sterility. A LAB reference (*L. plantarum* DSM 2648) was also used as a positive control in this study (Anderson et al., 2010).

### Lactobacillus cell surface characteristics

#### Hydrophobicity assay

Hydrophobicity of the LAB isolates was assessed via the microbial adhesion to hydrocarbons technique (Mishra & Prasad, 2005) using different hydrophobic solvents (toluene, xylene and hexadecane) (Sigma Aldrich, St. Louis, MO, USA). Results were reported as an average of three independent measurements, which were calculated according to Eq. (1).

(1) $$\rm{Hydrophobicity}\,(\%) = [(\rm{OD}_{\rm{before}} -\rm{OD}_{\rm{after}}) / \rm{OD}_{\rm{before}}] \times 100$$

where OD_before_ and OD_after_ represent the optical density before and after mixing with the hydrophobic solvents at OD_600_ nm.

#### Autoaggregation assay

The autoaggregation ability of the LAB isolates was evaluated as described by Angmo et al. (2016). The autoaggregation was measured at 4 h and 24 h and the percentage was reported as an average of three replicates according to Eq. (2).

(2) $${\rm Autoaggregation} =\left[1-\frac{A_t}{A_{0}}\right]\times100$$

where *A*_*t*_ represents the absorbance at time *t* and *A*_0_ the absorbance at *t* = 0.

#### Coaggregation assay

Coaggregation of LAB isolates against *Escherichia coli* (*E. coli*) and *Staphylococcus aureus* (*S. aureus*) was determined after 5 h incubation at 37 °C according to Pessoa et al. (2017). The coaggregation percentage was reported as an average of three independent measurements and was calculated using Eq. (3).

(3) $${\rm Coaggregation} = [(Ax + Ay)/2 - A(x + y)] / [(Ax + Ay)/2]$$

where *Ax* and *Ay* represents the absorbance of strains in the control tubes and *A*(*x + y*) represent the absorbance of the mixture.

#### Antimicrobial activity

The antimicrobial activity of cell-free culture supernatants of the LAB isolates was investigated by screening against *S. aureus* and *E. coli* (previously isolated from clinical cases of camel endometritis by Al-Fehaed (2014) as described by Mishra & Prasad (2005). Briefly, the LAB isolates were inoculated into MRS broth, grown overnight, and then centrifuged at 15,000×g for 20 min at 4 °C. Bacterial cells were discarded and the cell-free supernatants were neutralized and then passed through a 0.22 µm ministart filter (Sigma–Aldrich, Seelze, Germany). *S. aureus* and *E. coli* maintained on nutrient agar were sub-cultured in nutrient broth and incubated at 37 °C for 18 h. Bacterial cells were adjusted to 10^6^–10^7^ CFU of *S. aureus* and *E. coli* and coated Mueller Hinton agar plates (Oxoid, Basingstoke, UK). Wells (two mm) were formed in the plates and 50 µL of the supernatant were deposited in the wells. MRS broth medium (50 µL) has been used as a negative control. The plates were kept at room temperature for 1 h and then incubated at 37 °C for 24 h. An isolate with a clear inhibition zone of one mm or more was considered positive.

### Antibiotic susceptibility

Minimum inhibitory concentrations were determined using an E-test (BioMerieux, Lyon, France) for nine antibiotics: tetracycline, erythromycin, streptomycin, gentamicin, clindamycin, ampicillin, kanamycin, chloramphenicol and vancomycin. Concentrations for all antibiotics were 0.016–256 μg/mL, except for streptomycin at 0.064–1024 μg/mL. The LAB isolates were diluted to final concentrations of 10^6^–10^7^ CFU/mL and then inoculated onto Iso-Sensitest agar (Oxoid, Basingstoke, UK) supplemented with MRS agar (Georgieva et al., 2015), and incubated at 37 °C for 24 h. Breakpoint values were interpreted according to EFSA Panel on Additives and Products or Substances used in Animal Feed (FEEDAP) (2012) (Table S1).

### 16S rRNA amplification and sequencing

Genomic DNA was extracted from LAB isolates grown overnight in MRS broth using the QIAamp DNA mini-kit (Qiagen SA, Courtaboeuf, France). Extracted DNA was subjected to polymerase chain reaction (PCR) using primers 27F (5′-AGAGTTTGATCCTGGCTCAG-3′) and 1492R (5′TACGGYTACCTTGTTACGACTT-3′) specific for amplification of 16S rRNA (Klindworth et al., 2013). The PCR products were purified using the QIA quick PCR purification kit (Qiagen SA, Courtaboeuf, France) and then sequenced using an ABI 3500 Genetic analyzer (Applied Biosystems, Foster City, CA, USA).

The 16S rRNA gene sequence was analyzed using Geneious bioinformatics software (Version 11, available from http://www.geneious.com) and subjected to analysis via the National Center for Biological Information (NCBI) Basic Local Alignment Search Tool. The sequence was submitted to NCBI and accession numbers were obtained (Table S2). In order to construct a phylogenetic tree, the sequences and construction protocol was performed as previously described (Klaenhammer et al., 2005; Felis & Dellaglio, 2007).

To generate the sequence alignment, the retrieved sequences as described in Table S3 were aligned by MUSCLE add-on tool in Geneious package. During MUSCLE alignment two iterations were adopted comprising Kmer4_6 and pctid_kimura. The sequence-weighing scheme was set to CLUSTALW. For tree generation, previous report assured the lack of differences in tree construction after using distance matrix calculation (Kimura, Tamura 3 parameters) and tree reconstruction (neighbor joining and minimum evolution) (Felis & Dellaglio, 2007). In this work, the tree was generated after using Tamura genetic distance model, neighbor-joining tree build method and one thousand boost strap tree resampling. The tree was visualized by genedoc and Geneious software.

### Statistical analysis

Analysis was performed using R software (R Core Team, 2019, version 3.5.1, Vienna, Austria). One-way ANOVA was used to determine the significant differences between the LAB isolates. Tukey’s HSD test was used to perform multiple comparisons between the means. The significance level was set at a *P*-value of < 0.05.

## Results

### *Lactobacillus* isolation and characterization

Eighteen *Lactobacillus* isolates were isolated from the 75 vaginal swabs. Among the 18 LAB isolates, six (33.3%; MF1, MF2, MF4, MF5, MF6, and WD1) were *L. plantarum*, eight (44.4%; IG1, IG4, IG5, IG6, MF3, WD2, WD3, and WD4) were *L. fermentum* and four (22.2%; IG2, IG3, WD5 and WD6) were *L. rhamnosus*. All isolates were Gram-positive, rod-shaped and catalase-negative and were grown at pH 7.0, 4.0, 4.5, 3.5 and 3.0. However, none of the isolates showed hemolytic activity on sheep blood agar. Among all the tested LAB isolates, only four (22.2%) exhibited H_2_O_2_ production (Table 1).

**Table 1:** Hydrogen peroxide production andautoaggregation/coaggregation activity (mean ± standard error) of vaginal *Lactobacillus* isolates (*n* = 18) in dromedary camels.

Isolates		H_2_O_2_ production	Autoaggregation (%)		Coaggregation (%)	
ID	Species		4 h	24 h	*E. coli*	*S. aureus*
IG1	*L. fermentum*	Negative	7.8 ± 0.35^c^	25.9 ± 0.20^e^	11.4 ± 0.81^a^	14.3 ± 0.28^de^
IG2	*L. rhamnosus*	Positive	12.4 ± 0.46^a^	28.6 ± 0.46^d^	11.2 ± 0.78^ab^	18.1 ± 0.44^a^
IG3	*L. rhamnosus*	Negative	12.2 ± 0.76^a^	34.3 ± 0.49^b^	12.3 ± 0.17^a^	14.2 ± 0.38^de^
IG4	*L. fermentum*	Negative	3.9 ± 0.06^fg^	6.6 ± 1.47^g^	8.8 ± 0.31^c^	5.2 ± 0.08^h^
IG5	*L. fermentum*	Negative	12.4 ± 0.32^a^	38.1 ± 0.81^a^	3.4 ± 0.49^e^	7.3 ± 0.34^g^
IG6	*L. fermentum*	Negative	0.9 ± 0.25^ijk^	2.2 ± 0.26^h^	0^f^	0^i^
MF1	*L. plantarum*	Positive	5.8 ± 0.36^de^	29.0 ± 0.40^cd^	9.6 ± 0.47^bc^	16.5 ± 0.52^bc^
MF2	*L. plantarum*	Positive	9.5 ± 0.25^b^	25.0 ± 0.95^e^	10.7 ± 0.56^ab^	13.1 ± 0.76^e^
MF3	*L. fermentum*	Positive	5.1 ± 0.21^ef^	30.6 ± 0.46^c^	11.0 ± 0.44^ab^	16.7 ± 0.21^ab^
MF4	*L. plantarum*	Negative	1.7 ± 0.25^ij^	3.1 ± 0.15^h^	3.4 ± 1.59^e^	10.1 ± 0.89^f^
MF5	*L. plantarum*	Negative	6.2 ± 0.15^de^	9.7 ± 0.30^f^	3.7 ± 0.14^e^	4.4 ± 0.15^h^
MF6	*L. plantarum*	Negative	0^k^	1.6 ± 0.31^h^	0^f^	0^i^
WD1	*L. plantarum*	Negative	5.0 ± 0.10^ef^	10.9 ± 0.06^f^	1.4 ± 0.05^f^	4.0 ± 0.67^h^
WD2	*L. fermentum*	Negative	0.6 ± 0.21^jk^	2.9 ± 0.15^h^	0^f^	0^i^
WD3	*L. fermentum*	Negative	2.1 ± 0.32^hi^	2.8 ± 0.31^h^	6.2 ± 0.41^d^	15.3 ± 0.52^cd^
WD4	*L. fermentum*	Negative	6.4 ± 0.71^d^	6.8 ± 0.70^g^	8.4 ± 0.44^c^	9.3 ± 0.19^f^
WD5	*L. rhamnosus*	Negative	3.1 ± 0.15^gh^	9.2 ± 0.71^f^	8.1 ± 0.61^c^	4.8 ± 0.79^h^
WD6	*L. rhamnosus*	Negative	1.9 ± 0.87^hi^	2.9 ± 0.15^h^	0^f^	0^i^

**Note:**

Within columns, values marked by different letters (a–k) indicate differences in means (*P* < 0.05).

### *Lactobacillus* cell surface characteristics

**Figure 1:**
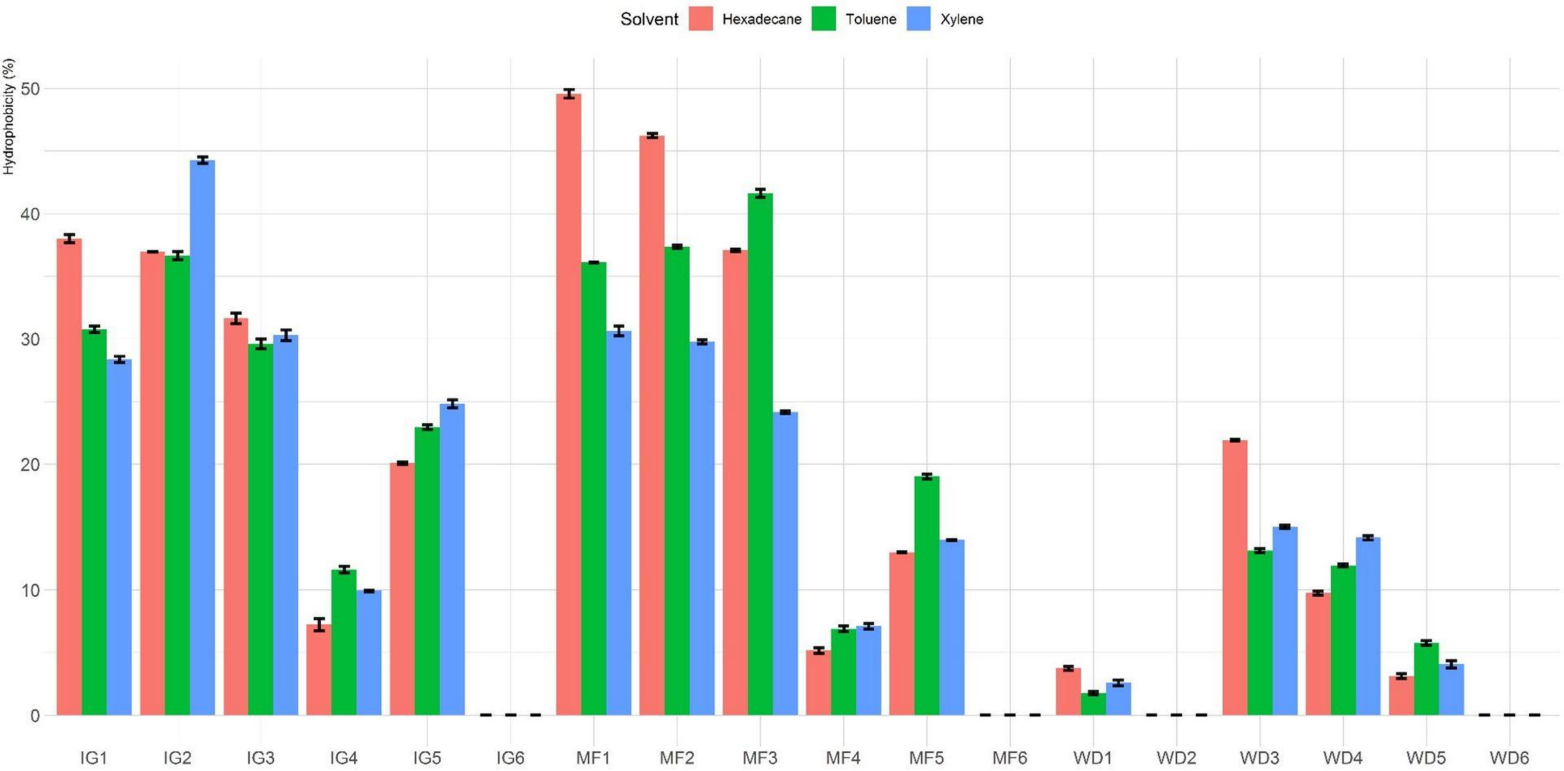
Hydrophobicity (%) of *Lactobacillus* isolates toward three different hydrophobic solvents (hexadecane, xylene and toluene).

The percentage of hydrophobicity of the LAB isolates is shown in Fig. 1. The percentages of hydrophobicity ranged from 0% to 49.6%, 0% to 44.3% and 0% to 41.6% for hexadecane, xylene and toluene, respectively. Among all the tested *Lactobacillus* isolates, MF1 showed maximum affinity towards hexadecane. Among the different hydrocarbons, maximum adhesion was seen with hexadecane (49.6 ± 0.6%), followed by xylene (44.3 ± 0.5%) and toluene (41.6 ± 0.6%). In general, isolates MF1, MF2, MF3, IG1, IG2 and IG3 showed higher hydrophobicity than the other tested isolates. Furthermore, four isolates (MF6, WD2, IG6 and WD6) exhibited no hydrophobicity toward the three hydrocarbons.

Percentages of autoaggregation and coaggregation are presented in Table 1. All isolates showed higher (*P* < 0.05) autoaggregation after 24 h of incubation compared to 4 h. Autoaggregation ability of the isolates after 4 h of incubation ranged from 0% (MF6) to 12.4% (IG2 and IG5), while after 24 h, isolates showed significant variability (*P* < 0.05) which ranged from 1.6% (MF6) to 38.1% (IG5). After 24 h of incubation, the LAB isolates MF1, MF2, MF3, IG1, IG2, IG3 and IG5 exhibited higher autoaggregation than the other isolates.

The coaggregation of the LAB isolates in the presences of *E. coli* and *S. aureus* is presented in Table 1. Fourteen of the 18 LAB isolates exhibited some coaggregation properties to *E. coli* and *S. aureus*. However, these isolates showed higher coaggregation (*P* < 0.05) toward *S. aureus* compared to *E. coli*. Five isolates exhibited the highest coaggregation toward *E. coli* (IG3 (12.3%), IG1 (11.4%), IG2 (11.2%), MF3 (11.0%), and MF2 (10.7%)), while eight isolates (IG2 (18.1%), MF3 (16.7%), MF1 (16.5%), WD3 (15.3%), IG1 (14.3%), IG3 (14.2%), MF2 (13.1%) and MF4 (10.1%)) showed high coaggregation activity against *S. aureus*. Overall, isolates IG2, MF3, IG3 and IG1 showed the highest coaggregation activity to both *E. coli* and *S. aureus*.

### Antimicrobial activity and antibiotic susceptibility

Table 2 presents the antimicrobial activity of cell-free culture supernatants against *S. aureus* and *E. coli* and the antibiotic resistance of the 18 LAB isolates against nine antibiotics. Six isolates (WD6, WD4, WD2, WD1, MF6 and MF4) showed no inhibitory activity against either *E. coli* or *S. aureus*, whereas all other isolates exhibited antimicrobial activity against both *S. aureus* and *E. coli*. These isolates showed greater antimicrobial activity against *S. aureus* compared to *E. coli*. LAB isolates displayed variation in susceptibility to different antibiotics (Table 2). All isolates were vancomycin resistant and sensitive to erythromycin, streptomycin, kanamycin and chloramphenicol. Three LAB isolates (IG1, IG4, WD4) were resistant to tetracycline.

**Table 2:** Antimicrobial activity and antibiotic susceptibilityof vaginal Lactobacillus isolates in dromedary camels.

Isolates		Antimicrobial activity		Antibiotic susceptibility (µg/mL)								
ID	*Species*	*E. coli*	*S. aureus*	AMP	STR	ERY	TET	KAN	GEN	ClI	CHL	VAN
IG1	*L. fermentum*	++	++	1	32	1	32	32	8	0.75	2	>256
IG2	*L. rhamnosus*	++	+++	0.85	16	0.075	1	16	1	0.035	2	>256
IG3	*L. rhamnosus*	+	++	0.95	8	0.45	0.5	8	0.55	0.85	3	>256
IG4	*L. fermentum*	–	+	0.75	16	0.035	16	8	2	2	2	>256
IG5	*L. fermentum*	++	+++	0.075	16	0.8	8	4	2	0.25	1	>256
IG6	*L. fermentum*	–	++	0.5	32	0.5	64	4	1	0.085	1	>256
MF1	*L. plantarum*	++	+++	0.5	16	1	24	16	8	0.075	4	>256
MF2	*L. plantarum*	++	+++	0.75	32	0.75	16	8	4	0.35	1	>256
MF3	*L. fermentum*	++	+++	1	32	1	4	32	8	0.9	4	>256
MF4	*L. plantarum*	–	–	1	16	0.75	64	32	32	0.25	8	>256
MF5	*L. plantarum*	+	++	0.02	4	0.075	8	16	4	0.75	2	>256
MF6	*L. plantarum*	–	–	3	16	0.75	16	32	2	0.25	1	>256
WD1	*L. plantarum*	–	–	2	8	0.25	8	50	2	0.019	8	>256
WD2	*L. fermentum*	–	–	3	8	0.75	16	16	8	0.75	2	>256
WD3	*L. fermentum*	++	+	4	4	0.75	4	4	1	0.5	0.5	>256
WD4	*L. fermentum*	–	–	0.75	8	0.75	9	4	4	0.95	2	>256
WD5	*L. rhamnosus*	+	+	1	4	0.5	2	16	0.95	0.075	3	>256
WD6	*L. rhamnosus*	–	–	2	2	0.95	16	4	0.025	0.5	2	>256

**Note:**

No inhibition (–), inhibition zone 1.0 to 2.0 mm (+), inhibition zone 2.1 to 4.0 mm (++), inhibition zone >4 mm (+++), ampicillin (AMP), streptomycin (STR), erythromycin (ERY), tetracycline (TET), kanamycin (KAN), gentamicin (GEN), clindamycin (CLI), chloramphenicol (CHL), vancomycin (VAN).

### Phylogenetic analysis

**Figure 2:**
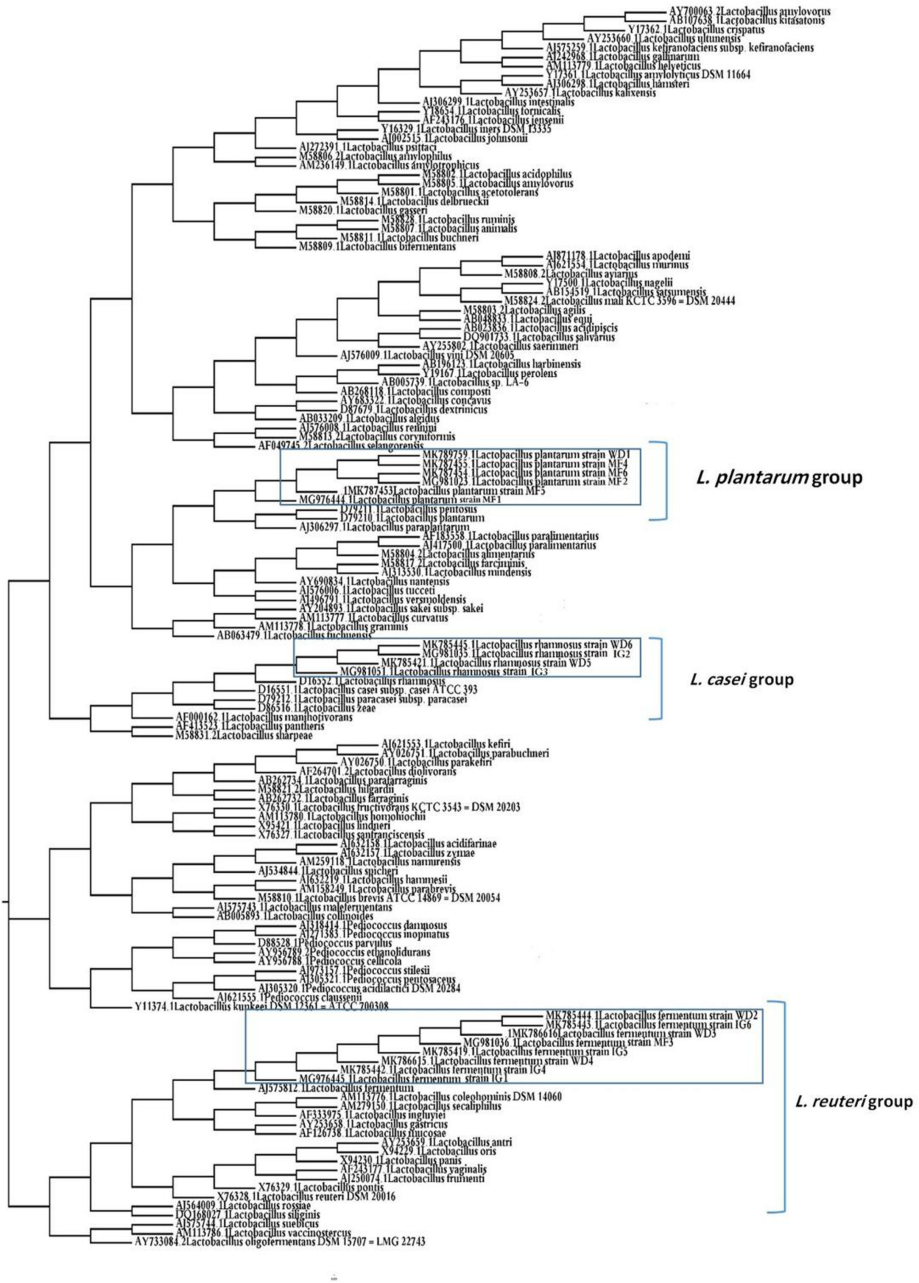
Neighbor-joining phylogenetic tree of the partial 16S rRNA gene sequences of isolated bacterial strains.

A phylogenetic tree of 18 LAB isolates was constructed based on the 16S rRNA sequences (Fig. 2). The LAB isolates were identified based on the highest hit scores (>99% sequence identity) and the sequences of all lactobacilli were deposited in the National Center for Biotechnology Information (NCBI) nucleotide sequence database. The accession numbers from GenBank for each isolate are presented in Table S2.

## Discussion

Vaginal microbiota are considered biomarker for the health of the female reproductive tract (Ravel et al., 2011; MacIntyre et al., 2015; Petrova et al., 2015) which is characterized by the presence of beneficial LAB (Tachedjian et al., 2017). Numerous studies have investigated the vaginal bacterial microflora of cows (Otero et al., 2000), mares (Fraga et al., 2008), sows (Bara et al., 1993), dogs (Noguchi, Tsukumi & Urano, 2003) and women (Mclean & Rosenstein, 2000). However, to the authors’ knowledge, information remains limited on vaginal microbiota of camels and the potential of LAB isolates to be used as probiotics in camels. Previous studies have documented the probiotic potential of LAB isolated from other animal species. For instances, in dairy cows, the use of LAB as probiotics lowered the incidence of infections in uterus and enhanced the local and systemic immune reactions of treated cows (Kummer et al., 1997; Deng et al., 2015). In this study, we hypothesized that isolation of LAB from camel vagina would also have probiotic potential and could be used for the treatment of many uterine infections, enhance local and systemic immune responses, and improve the health status of camel.

In this study, LAB were isolated from 24% (18/75) of cultured camel vaginal swabs, six isolates identified as *L. plantarum*, eight as *L. fermentum*, and four as *L. rhamnosus*. The same *Lactobacillus* spp. were isolated from vaginal samples of cattle (Otero et al., 2000), horses (Fraga et al., 2008) and humans (Juarez Tomas, Wiese & Nader-Macías, 2005). All isolates remained viable at both low (3.5 and 4.5) and high (7) pH levels, and thus the LAB isolates can survive in camel vagina during estrus (pH = 5.5–6.0) and pregnancy (pH = 7.0) phases (Nawito et al., 1967). The resistance of the isolates to pH varied depending on strains and species (Montville & Matthews, 2013). The results of this investigation were comparable to those of previous studies, where LAB were viable even after being exposed to low (2.0 and 3.0) and high (7.0) pH (Angmo et al., 2016; Abushelaibi et al., 2017).

Furthermore, in agreement with other studies, none of the isolated LAB showed hemolytic activity on sheep blood agar (Maragkoudakis et al., 2006; Hawaz, 2014). Resistance to gastric conditions and absence of hemolytic activity are important safety parameters for the selection of probiotic strains (FAO/WHO, 2002).

*Lactobacillus* plays an essential role in controlling the pathogen population in the vagina by producing antimicrobial compounds and competing with other pathogens for adherence to vaginal epithelium (Boris & Barbés, 2000). In this study, the probiotic potential of LAB isolates were assessed on microorganisms obtained from the same ecological niche because of the close relation between host specificity and colonization of indigenous microflora (Lin & Savage, 1984). Five in vitro assays, including H_2_O_2_ production, cell surface hydrophobicity, autoaggregation and coaggregation and antibacterial activity, were used to study the cell surface characteristics and bactericidal effects on pathogenic bacteria isolated from clinical field cases (Fraga et al., 2008).

Production of H_2_O_2_ is measured as one of the protection mechanisms of lactobacilli against vaginal infections (Pascual et al., 2006). Results of this study revealed that only four LAB isolates (22.2%) were H_2_O_2_-producing, which is lower than the 45% H_2_O_2_-producing vaginal lactobacilli isolated from cattle (Rodriguez et al., 2011) and the 96% (Eschenbach et al., 1989) and 62% (Pascual et al., 2006) isolated from women.

The results of LAB hydrophobicity in this investigation were comparable to those of other studies that reported <5‒47% hydrophobicity against hexadecane (Angmo et al., 2016), higher than the 22.2‒25% reported for LAB isolates from marine sources (Das, Khowala & Biswas, 2016) and lower than the 71‒100% reported for vaginal LAB isolates from women (Ocaña et al., 1999). The large differences in cell surface hydrophobicity of the LAB isolates in this study may have resulted from hydrophilic/hydrophobic extensions in the cell wall of the LAB isolates (Abushelaibi et al., 2017), the growth medium (Deepika et al., 2012) and environmental conditions (Ramiah, Van Reenen & Dicks, 2007), which could have affected the expression of surface proteins. In this study, maximum hydrophobicity was seen with hexadecane. However, Mishra & Prasad (2005) reported maximum hydrophobicity to octane although similar growth conditions were used in both studies.

Bacterial aggregation among microorganisms of the same strain (autoaggregation) or between genetically dissimilar strains (coaggregation) is considered as an important property of probiotics (Botes et al., 2008; Bao et al., 2010). Autoaggregation is essential in order to stimulate adhesion and colonization of probiotic microorganism in the urogenital and digestive tracts (Vandevoorde, Christiaens & Verstraete, 1992; Kos et al., 2003; Tomás et al., 2011). In this study, LAB isolates exhibited significantly higher autoaggregation after 24 h of incubation compared to 4 h. Abushelaibi et al. (2017) and Kumari et al. (2016) also reported that isolates of LAB showed significantly higher autoaggregation after 24 h compared to 3 h of incubation. The aggregation depends on incubation time and strains (Vanzieleghem et al., 2016; Rokana et al., 2017) and this may explain the broad variation in autoaggregation of the isolates of LAB used in this study.

Coaggregation abilities of LAB enable them to bind the pathogens and form a barrier that inhibits challenging bacteria from colonizing the mucous membranes (Ekmekci, Aslim & Ozturk, 2009). In this study, the majority of tested LAB isolates displayed some coaggregation properties with *S. aureus* and *E. coli* isolated from camels. However, the coaggregation percentages were higher with *S. aureus* compared to *E. coli*. Several studies have reported similar results and attributed the high coaggregation with *S. aureus* to its morphology (Collado, Meriluoto & Salminen, 2007; Botes et al., 2008). Furthermore, it was perceived that LAB isolates with high coaggregation percentages showed high autoaggregation, which is comparable to the results reported previously by Kumari et al. (2016).

One of the important properties that must be taken into account to consider for the selection of probiotic strains from the vagina in vitro is the suppression of pathogenic bacteria (Lepargneur & Rousseau, 2002). Antimicrobial properties of the cell-free supernatant of LAB tested against Gram-negative (*E. coli*) and Gram-positive (*S. aureus*) bacteria (isolated in our lab from cases suffered from endometritis) ranged from none to high antibacterial activity. These results were comparable with results previously reported by many authors (Angmo et al., 2016; Das, Khowala & Biswas, 2016; Zuo et al., 2016). Furthermore, the tested isolates showed higher activity against *S. aureus* than against *E. coli*, which is explained by the dependance of antimicrobial activity on pathogen species and strains (Zuo et al., 2016). The antimicrobial activity of LAB may be attributed to the bacteriocins produced by the majority of lactobacilli (Gillor, Nigro & Riley, 2005) and lactic acid which disrupt the outer membrane of the bacterial cell (Alakomi et al., 2000).

The antibiotic susceptibility of probiotics to commonly prescribed antibiotics is desirable (Kumari et al., 2016). In addition, the absence of transferable resistance genes is an imperative requirement for approval of probiotics (Danielsen & Wind, 2003). In this study, all isolates of LAB were susceptible to streptomycin, erythromycin, kanamycin and chloramphenicol and resistant to vancomycin. Three LAB isolates (IG1, IG4, WD4) were resistant to tetracycline. A similar lactobacilli antibiotic susceptibility profile stated by many researchers (Danielsen & Wind, 2003; Temmerman et al., 2003; Zoumpopoulou et al., 2008). Furthermore, DeLisle & Perl (2003) pointed out that resistance of LAB against a precise antibiotic may be due to the lack of the target site of that antibiotic on the LAB cells. For instance, vancomycin resistance was due to the presence of DAla-D-lactate in the LAB peptidoglycan instead of the normal dipeptide D-Ala-D-Ala, which is the target of the antibiotic (Coppola et al., 2005).

*Lactobacillus* isolates used as probiotics require an accurate taxonomic characterization (Reid et al., 2003). In the current study, the 16S rRNA amplification and sequences revealed that the tested LAB isolates were related to three *Lactobacillus* spp. (*L. fermentum*, *L. plantarum*, and *L. rhamnosus*). *L. fermentum* was the predominant species among the *Lactobacillus* isolates. Moreover, Otero et al. (2000) reported that *L. fermentum* was the prevalent species among the *Lactobacillus* isolates from the vagina of cows.

## Conclusions

Results showed that the cell free supernatant of some vaginal LAB isolates in camels, especially *L. plantarum* (MF1, MF2), *L. fermentum* (MF3) and *L. rhamnosus* (IG2), may have some in vitro probiotic properties against some of the common endometritis pathogens, however, the full probiotic potential of these specific isolates still requires further verification. Further investigation for the in vivo effect of these isolates is warranted.

## Supplemental Information

10.7717/peerj.8500/supp-1 Supplemental Information 1 Supplemental Tables 1–3.

## Additional Information and Declarations

### Data Availability

The following information was supplied regarding data availability: The minimal inhibitory concentration (MIC) cutoff values for Lactobacillus spp. according to EFSA, the Lactobacillus isolates identified by 16S rRNA gene sequencing, and their GenBank accession numbers are available in the Supplemental Tables.
