# Supplementary material for: Isolation and characterization of vaginal Lactobacillus spp. in dromedary camels (Camelus dromedarius): in vitro evaluation of probiotic potential of selected isolates
Source: PeerJ. 2020 Feb 5;8:e8500. doi: 10.7717/peerj.8500 (PMC7007732; doi:10.7717/peerj.8500)
Supplement: Supplemental Information 1 [file peerj-08-8500-s001.docx]

**Table 1**

Minimal inhibitory concentration (MIC) cut-off values for *Lactobacillus* spp. according to EFSA^1^

| **Antibiotics** | **MIC cut-off values(µg/mL) for *Lactobacillus* spp. according to EFSA** | | |
| --- | --- | --- | --- |
|  | ***Lactobacillus plantarum*** | ***Lactobacillus fermentum*** | ***Lactobacillus rhamnosus*** |
| **Ampicillin (AMP)** | 2 | 2 | 4 |
| **Streptomycin (STR)** | nr | 64 | 32 |
| **Erythromycin (ERY)** | 1 | 1 | 1 |
| **Tetracycline (TET)** | 32 | 8 | 8 |
| **Kanamicin (KAN)** | 64 | 32 | 64 |
| **Gentamicin (GEN)** | 16 | 16 | 16 |
| **Clindamycin (CLI)** | 1 | 1 | 1 |
| **Chlorfenicol (CHL)** | 8 | 4 | 4 |
| **Vancomycin (VAN)** | nr | nr | nr |

nr = not required.

**^1^** EFSA Panel on Additives and Products or Substances used in Animal Feed (FEEDAP), 2012. Guidance on the assessment of bacterial susceptibility to antimicrobials of human and veterinary importance. EFSA J. 10, 2740.

**Table 2**

LAB isolates identified by 16S rRNA gene sequencing and their Genbank accession numbers.

| **Isolate** | **Species** | **Accession No.** |
| --- | --- | --- |
| IG1 | *Lactobacillus fermentum* | [MG976445.1](https://www.ncbi.nlm.nih.gov/nuccore/MG976445.1?report=fasta) |
| IG2 | *Lactobacillus rhamnosus* | [MG981035.1](https://www.ncbi.nlm.nih.gov/nuccore/MG981035.1?report=fasta) |
| IG3 | *Lactobacillus rhamnosus* | [MG981051.1](https://www.ncbi.nlm.nih.gov/nuccore/MG981051.1?report=fasta) |
| IG4 | *Lactobacillus fermentum* | [MK785442](https://www.ncbi.nlm.nih.gov/nuccore/MK785442.1?report=fasta) |
| IG5 | *Lactobacillus fermentum* | [MK785419](https://www.ncbi.nlm.nih.gov/nuccore/MK785419.1?report=fasta) |
| IG6 | *Lactobacillus fermentum* | [MK785443](https://www.ncbi.nlm.nih.gov/nuccore/MK785443.1?report=fasta) |
| MF1 | *Lactobacillus plantarum* | [MG976444.1](https://www.ncbi.nlm.nih.gov/nuccore/MG976444.1?report=fasta) |
| MF2 | *Lactobacillus plantarum* | [MG981023.1](https://www.ncbi.nlm.nih.gov/nuccore/MG981023.1?report=fasta) |
| MF3 | *Lactobacillus fermentum* | [MG981036.1](https://www.ncbi.nlm.nih.gov/nuccore/MG981036.1?report=fasta) |
| MF4 | *Lactobacillus plantarum* | [MK787455](https://www.ncbi.nlm.nih.gov/nuccore/MK787455.1?report=fasta) |
| MF5 | *Lactobacillus plantarum* | [MK787453](https://www.ncbi.nlm.nih.gov/nuccore/MK787453.1?report=fasta) |
| MF6 | *Lactobacillus plantarum* | [MK787454](https://www.ncbi.nlm.nih.gov/nuccore/MK787454.1?report=fasta) |
| WD1 | *Lactobacillus plantarum* | [MK789759](https://www.ncbi.nlm.nih.gov/nuccore/MK789759.1?report=fasta) |
| WD2 | *Lactobacillus fermentum* | [MK785444](https://www.ncbi.nlm.nih.gov/nuccore/MK785444.1?report=fasta) |
| WD3 | *Lactobacillus fermentum* | [MK786616](https://www.ncbi.nlm.nih.gov/nuccore/MK786616.1?report=fasta) |
| WD4 | *Lactobacillus fermentum* | [MK786615](https://www.ncbi.nlm.nih.gov/nuccore/MK786615.1?report=fasta) |
| WD5 | *Lactobacillus rhamnosus* | [MK785421](https://www.ncbi.nlm.nih.gov/nuccore/MK785421.1?report=fasta) |
| WD6 | *Lactobacillus rhamnosus* | [MK785445](https://www.ncbi.nlm.nih.gov/nuccore/MK785445.1?report=fasta) |

**Table 3.**

The accession numbers and details of selected strains used in phylogenetic studies.

| **Name** | **Organism** | **Sequence Length** | **strain** | **Accession** | **Created Date** |
| --- | --- | --- | --- | --- | --- |
| Y19167 | Lactobacillus perolens | 1566 | L532 | Y19167.1 | Tue Aug 03 00:00:00 EEST 1999 |
| Y18654 | Lactobacillus fornicalis | 1421 | TV1018 | Y18654.1 | Fri Jun 16 00:00:00 EEST 2000 |
| Y17500 | Lactobacillus nagelii | 1100 | LuE10 | Y17500.1 | Thu Apr 13 23:00:00 EET 2000 |
| Y17362 | Lactobacillus crispatus | 1559 | DSM 20584 T | Y17362.1 | Tue Oct 27 23:00:00 EET 1998 |
| Y17361 | Lactobacillus amylolyticus DSM 11664 | 1558 | LA 5 | Y17361.1 | Tue Oct 27 23:00:00 EET 1998 |
| Y16329 | Lactobacillus iners DSM 13335 | 1539 | type strain: CCUG 28746 | Y16329.1 | Sun Jan 31 23:00:00 EET 1999 |
| Y11374 | Lactobacillus kunkeei | 1517 | YH-15 | Y11374.1 | Mon Mar 30 23:00:00 EET 1998 |
| X95421 | Lactobacillus lindneri | 1570 | DSM 20690 | X95421.1 | Tue Jul 15 00:00:00 EEST 1997 |
| X94230 | Lactobacillus panis | 1490 | DSM 6035T | X94230.1 | Fri May 10 00:00:00 EEST 1996 |
| X94229 | Lactobacillus oris | 1512 | DSM 4864T | X94229.1 | Fri May 10 00:00:00 EEST 1996 |
| X76330 | Lactobacillus fructivorans KCTC 3543 = DSM 20203 | 1570 | DSM 20203 T | X76330.1 | Fri Sep 02 00:00:00 EEST 1994 |
| X76329 | Lactobacillus pontis | 1570 | LTH 2587 | X76329.1 | Fri Sep 02 00:00:00 EEST 1994 |
| X76328 | Lactobacillus reuteri DSM 20016 | 1571 | DSM 20016 T | X76328.1 | Fri Sep 02 00:00:00 EEST 1994 |
| X76327 | Lactobacillus sanfranciscensis | 1572 | ATCC 27651 T | X76327.1 | Fri Sep 02 00:00:00 EEST 1994 |
| MG981051 | Lactobacillus rhamnosus | 950 | IG3 | MG981051.1 | Tue Feb 27 23:00:00 EET 2018 |
| MG981036 | Lactobacillus fermentum | 1050 | MF3 | MG981036.1 | Tue Feb 27 23:00:00 EET 2018 |
| MG981035 | Lactobacillus rhamnosus | 950 | IG2 | MG981035.1 | Tue Feb 27 23:00:00 EET 2018 |
| MG981023 | Lactobacillus plantarum | 980 | MF2 | MG981023.1 | Tue Feb 27 23:00:00 EET 2018 |
| MG976445 | Lactobacillus fermentum | 1050 | IG1 | MG976445.1 | Sun Feb 25 23:00:00 EET 2018 |
| MG976444 | Lactobacillus plantarum | 980 | MF1 | MG976444.1 | Sun Feb 25 23:00:00 EET 2018 |
| LSAF000162 | Lactobacillus manihotivorans | 1559 | OND 32; LMG 18010T | AF000162.1 | Mon Dec 07 23:00:00 EET 1998 |
| LBARR16SZ | Lactobacillus ruminis | 1567 |  | M58828.1 | Mon Apr 26 23:00:00 EET 1993 |
| LBARR16SV | Lactobacillus mali KCTC 3596 = DSM 20444 | 1502 | DSM 20444 | M58824.2 | Mon Apr 26 23:00:00 EET 1993 |
| LBARR16SS | Lactobacillus gasseri | 1521 |  | M58820.1 | Mon Apr 26 23:00:00 EET 1993 |
| LBARR16SQ | Lactobacillus hilgardii | 1521 | DSM 20176 | M58821.2 | Mon Apr 26 23:00:00 EET 1993 |
| LBARR16SP | Lactobacillus farciminis | 1515 | ATCC 29644 | M58817.2 | Mon Apr 26 23:00:00 EET 1993 |
| LBARR16SM | Lactobacillus delbrueckii | 1512 |  | M58814.1 | Mon Apr 26 23:00:00 EET 1993 |
| LBARR16SL | Lactobacillus coryniformis | 1516 | DSM 20001 | M58813.2 | Mon Apr 26 23:00:00 EET 1993 |
| LBARR16SJ | Lactobacillus buchneri | 1521 |  | M58811.1 | Mon Apr 26 23:00:00 EET 1993 |
| LBARR16SI | Lactobacillus brevis | 1569 | ATCC 14869 | M58810.1 | Mon Apr 26 23:00:00 EET 1993 |
| LBARR16SH | Lactobacillus bifermentans | 1574 |  | M58809.1 | Mon Apr 26 23:00:00 EET 1993 |
| LBARR16SG | Lactobacillus aviarius | 1504 | DSM 20655 | M58808.2 | Mon Apr 26 23:00:00 EET 1993 |
| LBARR16SF | Lactobacillus animalis | 1507 |  | M58807.1 | Mon Apr 26 23:00:00 EET 1993 |
| LBARR16SE | Lactobacillus amylophilus | 1516 | DSM 20533 | M58806.2 | Mon Apr 26 23:00:00 EET 1993 |
| LBARR16SD | Lactobacillus amylovorus | 1512 |  | M58805.1 | Mon Apr 26 23:00:00 EET 1993 |
| LBARR16SC | Lactobacillus alimentarius | 1514 | DSM 20249 | M58804.2 | Mon Apr 26 23:00:00 EET 1993 |
| LBARR16SB | Lactobacillus agilis | 1511 | DSM 20509 | M58803.2 | Mon Apr 26 23:00:00 EET 1993 |
| LBARR16SAC | Lactobacillus sharpeae | 1522 | DSM 20505 | M58831.2 | Mon Apr 26 23:00:00 EET 1993 |
| LBARR16SA | Lactobacillus acidophilus | 1569 |  | M58802.1 | Mon Apr 26 23:00:00 EET 1993 |
| LBARR16S | Lactobacillus acetotolerans | 1518 | DSM 20749 | M58801.1 | Mon Apr 26 23:00:00 EET 1993 |
| LBA16SRRNA | Lactobacillus paracasei subsp. paracasei | 1522 | JCM 8130 | D79212.1 | Thu Dec 14 23:00:00 EET 1995 |
| LBA16SRRN2 | Lactobacillus pentosus | 1519 |  | D79211.1 | Thu Dec 14 23:00:00 EET 1995 |
| LBA16SRRN1 | Lactobacillus plantarum | 1519 | JCM 1149 | D79210.1 | Thu Dec 14 23:00:00 EET 1995 |
| DQ901733 | Lactobacillus salivarius | 1063 | DSM 20555 | DQ901733.1 | Fri Oct 06 23:00:00 EET 2006 |
| DQ168027 | Lactobacillus siliginis | 1310 | M1-212 | DQ168027.1 | Thu Oct 27 23:00:00 EET 2005 |
| D88528 | Pediococcus parvulus | 1447 | JCM5889 | D88528.1 | Fri Nov 01 23:00:00 EET 1996 |
| D87679 | Lactobacillus dextrinicus | 1464 | JCM 5887 | D87679.1 | Thu Sep 12 00:00:00 EEST 1996 |
| D86516 | Lactobacillus zeae | 1522 | ATCC 15820 | D86516.1 | Tue Feb 18 23:00:00 EET 1997 |
| D16552 | Lactobacillus rhamnosus | 1521 |  | D16552.1 | Fri Feb 21 23:00:00 EET 1997 |
| D16551 | Lactobacillus casei subsp. casei | 1522 | JCM 1134 | D16551.1 | Fri Feb 21 23:00:00 EET 1997 |
| AY956789 | Pediococcus ethanolidurans | 1501 | Z-9 | AY956789.2 | Sat Apr 02 23:00:00 EET 2005 |
| AY956788 | Pediococcus cellicola | 1542 | Z-8 | AY956788.1 | Sat Apr 02 23:00:00 EET 2005 |
| AY733084 | Lactobacillus oligofermentans | 1562 | AMKR18 | AY733084.2 | Wed Sep 29 00:00:00 EEST 2004 |
| AY700063 | Lactobacillus amylovorus | 1565 | OTU171-001 | AY700063.2 | Sun Jan 15 23:00:00 EET 2006 |
| AY690834 | Lactobacillus nantensis | 1561 | LP33 | AY690834.1 | Thu Apr 21 23:00:00 EET 2005 |
| AY683322 | Lactobacillus concavus | 1528 | AS 1.5017 | AY683322.1 | Sun Aug 29 00:00:00 EEST 2004 |
| AY255802 | Lactobacillus saerimneri | 1568 | GDA154 (T); LMG 22087 (T); DSM 16049 (T); CCUG 48462 (T) | AY255802.1 | Tue Apr 22 23:00:00 EET 2003 |
| AY253660 | Lactobacillus ultunensis | 1550 | Kx146C1; LMG 22117T; DSM 16047T; CCUG 48460T | AY253660.1 | Sat Apr 26 00:00:00 EEST 2003 |
| AY253659 | Lactobacillus antri | 1520 | Kx146A4; LMG 22111T; DSM 16041T; CCUG 48456T | AY253659.1 | Sat Apr 26 00:00:00 EEST 2003 |
| AY253658 | Lactobacillus gastricus | 1550 | Kx156A7; LMG 22113T; DSM 16045T; CCUG 48454T | AY253658.1 | Sat Apr 26 00:00:00 EEST 2003 |
| AY253657 | Lactobacillus kalixensis | 1555 | Kx127A2; LMG 22115T; DSM 16043T; CCUG 48459T | AY253657.1 | Sat Apr 26 00:00:00 EEST 2003 |
| AY204893 | Lactobacillus sakei subsp. sakei | 1406 | DSM 20017 | AY204893.1 | Fri Mar 07 23:00:00 EET 2003 |
| AY026751 | Lactobacillus parabuchneri | 1219 | LMG 11457T | AY026751.1 | Sat Mar 03 23:00:00 EET 2001 |
| AY026750 | Lactobacillus parakefiri | 1396 | LMG 15133T | AY026750.1 | Sat Mar 03 23:00:00 EET 2001 |
| AM279150 | Lactobacillus secaliphilus | 1551 | type strain: TMW 1.1309 | AM279150.1 | Fri Oct 06 23:00:00 EET 2006 |
| AM259118 | Lactobacillus namurensis | 1518 | type strain: LMG 23583 = R-27965 | AM259118.1 | Tue Oct 10 23:00:00 EET 2006 |
| AM236149 | Lactobacillus amylotrophicus | 1488 | type strain: LMG 11400 | AM236149.1 | Sun Nov 05 23:00:00 EET 2006 |
| AM158249 | Lactobacillus parabrevis | 1518 | type strain: LMG 11984 | AM158249.1 | Wed Apr 26 23:00:00 EET 2006 |
| AM113786 | Lactobacillus vaccinostercus | 1553 | type strain:DSM 20634 | AM113786.1 | Wed Oct 12 23:00:00 EET 2005 |
| AM113780 | Lactobacillus homohiochii | 1537 | type strain:DSM 20571 | AM113780.1 | Wed Oct 12 23:00:00 EET 2005 |
| AM113779 | Lactobacillus helveticus | 1554 | type strain:DSM 20075 | AM113779.1 | Wed Oct 12 23:00:00 EET 2005 |
| AM113778 | Lactobacillus graminis | 1548 | type strain:DSM 20719 | AM113778.1 | Wed Oct 12 23:00:00 EET 2005 |
| AM113777 | Lactobacillus curvatus | 1559 | type strain:DSM 20019 | AM113777.1 | Wed Oct 12 23:00:00 EET 2005 |
| AM113776 | Lactobacillus coleohominis | 1564 | type strain:DSM 14060 | AM113776.1 | Wed Oct 12 23:00:00 EET 2005 |
| AJ973157 | Pediococcus stilesii | 1529 | type strain: LMG 23082 = FAIR-E 180 | AJ973157.1 | Wed Feb 01 23:00:00 EET 2006 |
| AJ871178 | Lactobacillus apodemi | 1532 | type strain: ASB1 | AJ871178.1 | Wed Dec 22 23:00:00 EET 2004 |
| AJ632219 | Lactobacillus hammesii | 1549 | type strain: TMW 1.1236 | AJ632219.1 | Wed Oct 27 23:00:00 EET 2004 |
| AJ632158 | Lactobacillus acidifarinae | 1518 | type strain: LMG 22200 = R-19065 | AJ632158.1 | Thu Nov 04 23:00:00 EET 2004 |
| AJ632157 | Lactobacillus zymae | 1518 | type strain: LMG 22198 = R-18615 | AJ632157.1 | Thu Nov 04 23:00:00 EET 2004 |
| AJ621555 | Pediococcus claussenii | 1472 | type strain: DSM 14800 | AJ621555.1 | Mon Mar 07 23:00:00 EET 2005 |
| AJ621554 | Lactobacillus murinus | 1459 | type strain: LMG 14189 | AJ621554.1 | Thu Jun 30 00:00:00 EEST 2005 |
| AJ621553 | Lactobacillus kefiri | 1456 | type strain: LMG 9480 | AJ621553.1 | Thu Jun 30 00:00:00 EEST 2005 |
| AJ576009 | Lactobacillus vini | 1503 | type strain: CECT 5924 | AJ576009.1 | Wed Jul 16 00:00:00 EEST 2003 |
| AJ576008 | Lactobacillus rennini | 1544 | CECT 5923 | AJ576008.1 | Wed Jul 16 00:00:00 EEST 2003 |
| AJ576006 | Lactobacillus tucceti | 1557 | CECT 5920 | AJ576006.1 | Wed Jul 16 00:00:00 EEST 2003 |
| AJ575812 | Lactobacillus fermentum | 1555 | CECT 562 | AJ575812.1 | Wed Jul 09 00:00:00 EEST 2003 |
| AJ575744 | Lactobacillus suebicus | 1573 | CECT 5917T | AJ575744.1 | Tue Jul 08 00:00:00 EEST 2003 |
| AJ575743 | Lactobacillus malefermentans | 1525 | CECT 5928T | AJ575743.1 | Tue Jul 08 00:00:00 EEST 2003 |
| AJ575259 | Lactobacillus kefiranofaciens subsp. kefiranofaciens | 1516 | type strain: LMG 19149 | AJ575259.1 | Sun Oct 19 23:00:00 EET 2003 |
| AJ564009 | Lactobacillus rossiae | 1368 | CS1 | AJ564009.1 | Fri Jun 20 00:00:00 EEST 2003 |
| AJ534844 | Lactobacillus spicheri | 1536 | type strain: LTH 5753 | AJ534844.1 | Sun Nov 30 23:00:00 EET 2003 |
| AJ496791 | Lactobacillus versmoldensis | 1610 | type strain: KU-3 | AJ496791.1 | Tue Jul 30 00:00:00 EEST 2002 |
| AJ417500 | Lactobacillus paralimentarius | 1563 | DSM 13238 | AJ417500.1 | Sun Oct 28 23:00:00 EET 2001 |
| AJ318414 | Pediococcus damnosus | 1561 | DSM 20331 | AJ318414.1 | Tue Jun 26 00:00:00 EEST 2001 |
| AJ313530 | Lactobacillus mindensis | 1544 | TMW 1.80 | AJ313530.1 | Fri Nov 30 23:00:00 EET 2001 |
| AJ306299 | Lactobacillus intestinalis | 1513 | DSM 6629T | AJ306299.1 | Thu Feb 08 23:00:00 EET 2001 |
| AJ306298 | Lactobacillus hamsteri | 1510 | DSM 5661T | AJ306298.1 | Thu Feb 08 23:00:00 EET 2001 |
| AJ306297 | Lactobacillus paraplantarum | 1502 | DSM 10667T | AJ306297.1 | Wed Feb 07 23:00:00 EET 2001 |
| AJ305321 | Pediococcus pentosaceus | 1569 | DSM 20336 (T) | AJ305321.1 | Thu Feb 08 23:00:00 EET 2001 |
| AJ305320 | Pediococcus acidilactici | 1569 | DSM 20284 (T) | AJ305320.1 | Thu Feb 08 23:00:00 EET 2001 |
| AJ272391 | Lactobacillus psittaci | 1421 |  | AJ272391.1 | Fri May 18 00:00:00 EEST 2001 |
| AJ271383 | Pediococcus inopinatus | 1551 | type strain DSM 20285 | AJ271383.1 | Tue Feb 01 23:00:00 EET 2000 |
| AJ250074 | Lactobacillus frumenti | 1561 | TMW 1.666 | AJ250074.1 | Thu Mar 02 23:00:00 EET 2000 |
| AJ242968 | Lactobacillus gallinarum | 1533 | ATCC 33199 | AJ242968.1 | Sat Jun 19 00:00:00 EEST 1999 |
| AJ002515 | Lactobacillus johnsonii | 1487 | ATCC 33200 (T) | AJ002515.1 | Mon Nov 03 23:00:00 EET 1997 |
| AF413523 | Lactobacillus pantheris | 1559 |  | AF413523.1 | Thu Sep 27 00:00:00 EEST 2001 |
| AF333975 | Lactobacillus ingluviei | 1506 | KR3 | AF333975.1 | Mon Jul 02 00:00:00 EEST 2001 |
| AF264701 | Lactobacillus diolivorans | 1491 | JKD6 | AF264701.2 | Sun May 28 00:00:00 EEST 2000 |
| AF243177 | Lactobacillus vaginalis | 1541 | ATCC 49540 | AF243177.1 | Wed Apr 19 23:00:00 EET 2000 |
| AF243176 | Lactobacillus jensenii | 1496 | ATCC 25258 | AF243176.1 | Wed Apr 19 23:00:00 EET 2000 |
| AF183558 | Lactobacillus paralimentarius | 1522 | AP1077 | AF183558.1 | Fri Dec 31 23:00:00 EET 1999 |
| AF126738 | Lactobacillus mucosae | 1568 | CCUG 43179 (T); S32; DSM 13345 | AF126738.1 | Thu Jun 24 00:00:00 EEST 1999 |
| AF049745 | Lactobacillus selangorensis | 1495 | LMG 17710 | AF049745.2 | Fri Jan 01 23:00:00 EET 1999 |
| AB268118 | Lactobacillus composti | 1522 | NRIC 0689 | AB268118.1 | Mon Dec 11 23:00:00 EET 2006 |
| AB262734 | Lactobacillus parafarraginis | 1556 | NRIC 0677 | AB262734.1 | Mon Nov 27 23:00:00 EET 2006 |
| AB262732 | Lactobacillus farraginis | 1556 | NRIC 0678 | AB262732.1 | Mon Nov 27 23:00:00 EET 2006 |
| AB196123 | Lactobacillus harbinensis | 1507 | SBT10908 | AB196123.1 | Wed Sep 28 00:00:00 EEST 2005 |
| AB154519 | Lactobacillus satsumensis | 1556 | NRIC 0604 | AB154519.1 | Sat Sep 18 00:00:00 EEST 2004 |
| AB107638 | Lactobacillus kitasatonis | 1506 | JCM 1039 | AB107638.1 | Wed Apr 23 23:00:00 EET 2003 |
| AB063479 | Lactobacillus fuchuensis | 1329 | JCM 11249 | AB063479.1 | Thu Dec 20 23:00:00 EET 2001 |
| AB048833 | Lactobacillus equi | 1432 | YIT 0455 | AB048833.1 | Tue Sep 19 00:00:00 EEST 2000 |
| AB033209 | Lactobacillus algidus | 1505 | JCM 10491 | AB033209.1 | Fri Mar 24 23:00:00 EET 2000 |
| AB023836 | Lactobacillus acidipiscis | 1406 | FS60-1 | AB023836.1 | Sat Jun 24 00:00:00 EEST 2000 |
| AB005893 | Lactobacillus collinoides | 1549 | JCM1123 | AB005893.1 | Fri Jul 25 00:00:00 EEST 1997 |
| AB005739 | Lactobacillus sp. | 1555 | LA-6 | AB005739.1 | Tue Jul 22 00:00:00 EEST 1997 |
